# Interdecadal variability in pan-Pacific and global SST, revisited

**DOI:** 10.1007/s00382-018-4240-1

**Published:** 2018-05-21

**Authors:** Ka-Kit Tung, Xianyao Chen, Jiansong Zhou, King-Fai Li

**Affiliations:** 1 Department of Applied Mathematics, University of Washington, Seattle, WA USA; 2 Physical Oceanography Laboratory/CIMST, Ocean University of China, and Qingdao National Laboratory of Marine Science and Technology, Qingdao, 266100 China; 3 Department of Environmental Science, University of California, Riverside, CA USA

## Abstract

Interest in the “Interdecadal Pacific Oscillation (IPO)” in the global SST has surged recently on suggestions that the Pacific may be the source of prominent interdecadal variations observed in the global-mean surface temperature possibly through the mechanism of low-frequency modulation of the interannual El Nino-Southern Oscillation (ENSO) phenomenon. IPO was defined by performing empirical orthogonal function (EOF) analysis of low-pass filtered SST. The low-pass filtering creates its unique set of mathematical problems—in particular, mode mixing—and has led to some questions, many unanswered. To understand what these EOFs are, we express them first in terms of the recently developed pairwise rotated EOFs of the unfiltered SST, which can largely separate the high and low frequency bands without resorting to filtering. As reported elsewhere, the leading rotated dynamical modes (after the global warming trend) of the unfiltered global SST are: ENSO, Pacific Decadal Oscillation (PDO), and Atlantic Multidecadal Oscillation (AMO). IPO is not among them. The leading principal component (PC) of the low-pass filtered global SST is usually defined as IPO and it is seen to comprise of ENSO, PDO and AMO in various proportions depending on the filter threshold. With decadal filtering, the contribution of the interannual ENSO is understandably negligible. The leading dynamical mode of the filtered global SST is mostly AMO, and therefore should not have been called the Interdecadal “Pacific” Oscillation. The leading dynamical mode of the filtered pan-Pacific SST is mostly PDO. This and other low-frequency variability that have the action center in the Pacific, from either the pan-Pacific or global SST, have near zero global mean.

## Introduction

In the classic 1997 paper entitled “ENSO-like Interdecadal Variability, 1900–1993”, Zhang, Wallace and Battisti (hereafter ZWB) studied in detail the leading dynamical EOF of monthly SST*, i.e. the “SST deviation”, defined as the SST field with the concurrent global mean SST subtracted from each grid point. By employing highpass and lowpass Fourier filters with a 6-year cutoff, the authors noted that its PC is a combination of the usual interannual variation associated with ENSO, which they called “ENSO cycle”, and an interdecadal frequency, whose spatial pattern they described as “ENSO-like”. The latter term was defined by ZWB as “a spatial pattern similar to the interannual variability-related pattern except that the meridional scale of tropical anomalies is broader at the decadal time scale”. An update with 20 more years of data can be found in Chen and Wallace (2015). The nature of this frequency combination is further clarified in Chen and Wallace (2016) (hereafter CW16) for pan-Pacific SST* (100°E–80°W, 65°S–65°E), and by Chen et al. (2017) (hereafter CWT17) for global SST, as “mode mixing”: In conventional EOF analysis, the PC times series are required to be uncorrelated, and the EOFs (the spatial pattern associated with the PC) are required to be orthogonal to each other. Since there are very few large-scale physical modes that satisfy this twin-orthogonality constraint, the EOF analysis often breaks them into various pieces and reconfigures them in various combinations to conform to the mathematical constraint. Following Takahashi et al. (2011), CW16 added and subtracted PCs of the two leading dynamical modes in pan-Pacific SST*. The operation effectively separated the interannual frequencies from the lower-frequencies (decadal and interdecadal). The interannual frequency band, at 2–7 years, is associated with the “ENSO cycle”, with a more tightly focused eastern equatorial Pacific warm tongue with the maximum amplitude off the coast of Americas, and “devoid of extratropical structure” (CW16). This mode will be called here simply as ENSO. The lower-frequency mode, which was termed the *P* mode in CW16 and the PDO mode in CWT17, has almost the same spatial pattern as the traditionally defined PDO (as the leading dynamical EOF of the Pacific SST north of 20°N) (Mantua et al. 1997) in the region of their overlap. One notable feature of the pan-Pacific PDO (CW16) or the global PDO (CWT17) is that its center of action is in the North Pacific, not in the tropical Pacific. There is not much overlap in its spatial variance with ENSO in the Nino 1–2 region in the equatorial region, although it has some amplitude of opposite sign in the central equatorial Pacific, in the Nino 4 region. Additionally, the spectrum of the PDO mode is broad, but lowpass filtering with threshold above 10 years yields increasingly small variances over the tropical Pacific (as will be shown). The method of pairwise rotation effectively separated the two frequency bands, and remedied the “mode mixing” problem of the conventional EOF analysis, without resorting to filtering. This result then forms the basis for us now to understand the low-pass filtered results that are now commonly used.

During the intervening two decades, various other methods have been tried to separate these two frequency bands in the leading dynamical PC of the global SST. The importance of this task was probably attributed to the common notion that the decadal and interdecadal frequencies are part of ENSO phenomenon. The lower-frequency part of the PC was described by some authors as the “interdecadal modulation of ENSO”. However, the phenomenon of nonlinear modulation cannot be easily studied using the EOF analysis, which is a linear statistical method. The low-frequency variation that was often characterized as modulation of the interannual ENSO is dominated instead by a linear superimposition of frequency bands and can be easily separated into two uncorrelated modes. We shall review the previous approaches.

These previous approaches involved either filtering of the PC after an EOF analysis, or the filtering of the SST before doing the EOF analysis. Mathematically, the operations of EOF expansion and filtering do not commute. The spatial pattern of the leading mode is the pattern that maximizes the variance of the SST, and that of the second mode maximizes the remaining variance while being orthogonal to the first mode, and so on to the higher modes. In the case of relevance, the reason the leading dynamical PC is “leading” is because of its high variance in the interannual frequencies (associated with ENSO-cycle). When the interannual frequencies are filtered out, the remaining decadal frequency band in this leading PC, being of lower variance, should have been relegated to the higher PCs, and its associated spatial pattern may not bear any resemblance to ENSO-cycle like features associated with this leading PC.

The low-frequency dynamics can be studied more systematically by first low-pass filtering the SST and then performing the EOF analysis. This mathematically correct approach was adopted in the usual definition of the Interdecadal Pacific Oscillation (IPO), starting from Folland et al. (1999). However, the downside (compared with rotated EOF analysis) is that there is a drastic reduction of the degrees of freedom in the resulting SST after decadal low-pass filtering. For the 13.3 years low-pass filter applied to the 84 years span of SST by Folland et al. (1999), one can give a simplistic estimate of the degrees of freedom as 84/13.3~6. This is even likely a gross overestimate because autocorrelation is not taken into account. The low degrees of freedom lead to a number of sensitivities. These include sensitivity to the way the data are processed as well as to the time span used, and such sensitivity needs to be recognized even if it cannot be remedied. In particular, we will point out that the strong variance in the equatorial eastern Pacific in IPO defined by some authors arises from one such sensitivity to using data before 1910.

Another sensitivity is to the threshold *n* of the low-pass filter. For pan-Pacific SST*, ZWB used *n* = 6 years for the threshold of their low-pass filter. It can be shown that the spatial pattern similar to ENSO variability gradually disappears in the tropical Pacific for *n* larger than 8 years. For global SST under decadal low-pass filtering, the center of action of IPO shifts to the Atlantic, taking on the spatial shape close to that of the Atlantic Multidecadal Oscillation (AMO). So this IPO is “Pacific” in name only, but this fact is often not noticed when only the PC is studied.

There are other ways for defining IPO so that its center of action remains in the Pacific basin. These include using only the pan-Pacific SST*, as we will do here, or pan-Pacific SST, or defining IPO as the third EOF of the decadally filtered global SST, or bypassing the EOF analysis altogether by defining IPO as differences of mean SSTs in some regions of the Pacific. We will review these as well and point out that these forms of IPO do not contribute to the global mean SST or global mean surface temperature, because they are comprised mostly of PDO, and PDO contributes little to the global mean SST or global mean land plus ocean surface temperature [as shown in Chen and Tung (2017)].

In a number of recent studies of the contribution of natural climate variability to the recent hiatus in global warming, IPO has been used to characterize the dominant mode of natural variability on the interdecadal time scale in the Pacific sector (Dai et al. 2015; England et al. 2014; Kosaka and Xie 2016; Meehl et al. 2013). The recent two-decade long intensification of the Pacific trade winds has also been attributed to IPO, although some of the same authors also raised concerns about this interpretation (England et al. 2014; Han et al. 2014; McGregor et al. 2014). Two appealing aspects of IPO have often been alluded to: (1) the phase (in particular, the zero crossings) of IPO appears to coincide with the “climate regime shifts” in the multidecadal global-mean surface temperature variation, and (2) its “ENSO-like” spatial pattern implicates the tropical Pacific (Dai et al. 2015; England et al. 2014; Kosaka and Xie 2016). We will discuss whether these attributes of IPO are robust.

## Leading EOF in pan-Pacific SST*

**Fig. 1:**
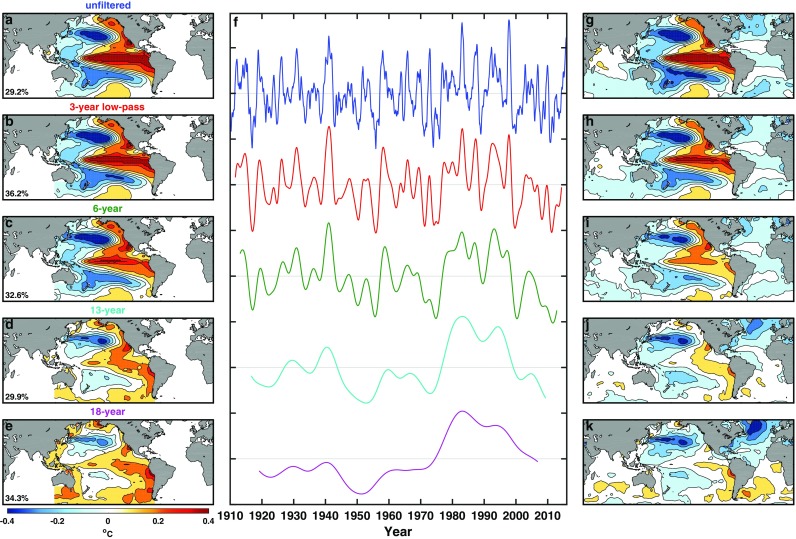
Leading EOF and PC of pan-Pacific SST*. Leading EOF (left column) and PC (middle column) of unfiltered (top row) and low-pass filtered (lower rows, with the various value of low pass *n* indicated) pan-Pacific SST*. Right column: global spatial pattern obtained by regressing corresponding filtered global SST* onto the pan-Pacific PCs in the middle column

ZWB performed an EOF analysis on the SST*. It supposedly removes the influence of global warming, which is dominant in most SST fields. The leading EOF and PC for the unfiltered data are shown in the top row of Fig. 1. The lower rows are the corresponding EOF and PC for the filtered SST*.

As mentioned in the Introduction, the unfiltered leading PC is a mix between the interannual frequencies of canonical ENSO and the decadal and interdecadal frequencies of PDO. ZWB’s “ENSO-like interdecadal variability” is shown in the third row, which is obtained from 6-year low-pass filtered SST*. The “ENSO-like” feature refers to the tropical cold tongue, which is broader than that associated with ENSO-cycle, and extends into the extratropics to the northeast and southeast. As the threshold of the filter increases beyond *n* = 8, the variance in the tropical eastern Pacific gradually diminishes, showing the weakness of tropical Pacific’s interdecadal variability. As *n* increases to a decade or longer, Atlantic would have emerged as the dominant region of variability if the global SST were used, as will be shown in Sect. 3. Some authors prefer to project global SST onto the leading PC obtained from an EOF analysis of pan-Pacific SST*. Such a “global” spatial pattern, shown in the right column of Fig. 1, is still dominated by the Pacific, but this is due to the bias towards the Pacific when the Pacific PC is used in the regression, which is actually a non-orthogonal time series decomposition because the Pacific PC is not a component of the orthogonal basis of the global EOF decomposition. The correct global pattern should be obtained by performing EOF analysis of the global SST.

## Leading dynamical EOF of low-pass filtered global SST

**Fig. 2:**
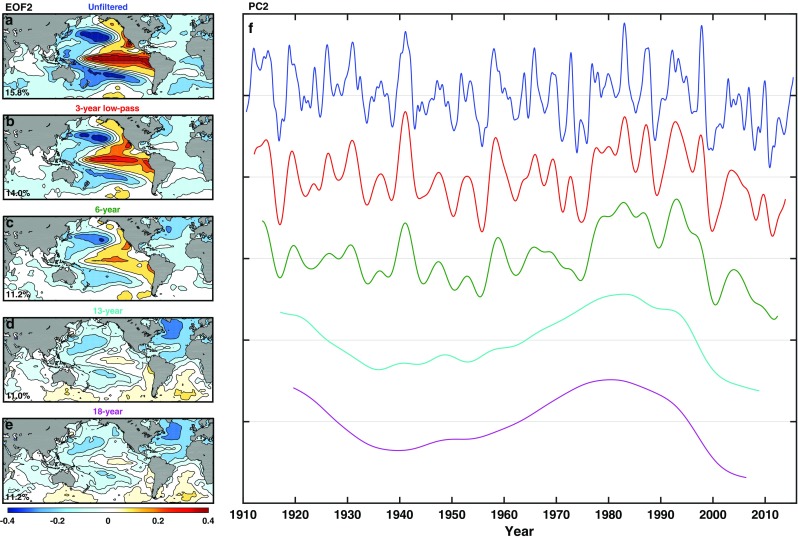
Leading dynamical EOF and PC of global SST. Leading dynamical EOF (left column) and PC (right column) of the low-passed global SST for various low-pass filter threshold

Figure 2 shows the second EOF/PC of the global SST under various low-pass filtering. The second EOF is the leading dynamical mode, since the first EOF is the global warming trend. Conventional EOF decomposition is used. For small *n*, the leading dynamical mode of the global SST is very close to the leading dynamical mode of the pan-Pacific SST* of ZWB and Chen and Wallace (2015). This is because the interannual ENSO phenomenon in the Pacific dominates the global variance. For *n* = 6, the “ENSO-like” variability of ZWB can be seen, but an Atlantic pattern is emerging. For *n* > 10, the interannual ENSO is filtered out; AMO in the Atlantic basin now has the larger variance as the tropical Pacific’s variability resembling ENSO is disappearing. So the leading EOF of decadally filtered global SST is mostly AMO, with some slight contribution by PDO in the northwestern Pacific.

## A brief historical review of IPO

The earliest mention of IPO appears to be Power et al. (1999) in a paper entitled “Inter-decadal modulation of the impact of ENSO on Australia”, which refers to a technical report by Folland et al. (1999). Folland et al. (1999) were interested in examining the “near bidecadal” variations in the Pacific. A 13.3-year low-pass filter was used to “remove ENSO-related variability and to concentrate on the near bidecadal time scale and its longer-term modulation”. As a consequence, their IPO does not have ENSO-related variability in the eastern tropical Pacific that some other authors referred to as “ENSO-like”. As originally defined by Folland et al. 1999 and Power et al. (1999), IPO is the third EOF of the low-pass global SST. They noted a “modest peak around 30 years” in its spectrum, and “the weights over the North west Pacific exceed in magnitude those of opposite sign over the tropical east Pacific”, and differs from ENSO by “the near zero weights over the easternmost Tropical Pacific”. From hindsight (with the recent perspective gained from the pan-Pacific modes of CW16), these patterns bear more resemblance to the pan-Pacific PDO than to the tropical ENSO-related features that later authors ascribed to IPO and its role in modulating the interannual ENSO.

Later Parker et al. (2007) used an 11-year low-pass filter and redefined the second EOF of the low-pass global SST as IPO, a definition that is currently adopted. Its spatial pattern has what appears to be a more prominent ENSO-related variability in the tropical eastern Pacific. It is puzzling since the 11-year low-pass filter should have been long enough in removing the interannual ENSO. It turns out to be a data quality and sensitivity problem, as will be discussed later. Its associated PC, which is available online, is often adopted as IPO index. The low-pass threshold has evolved from 13.3 years (Folland et al. 1999) to 11 years (Parker et al. 2007) and recently back to 13 years (Henley et al. 2015).

**Fig. 3:**
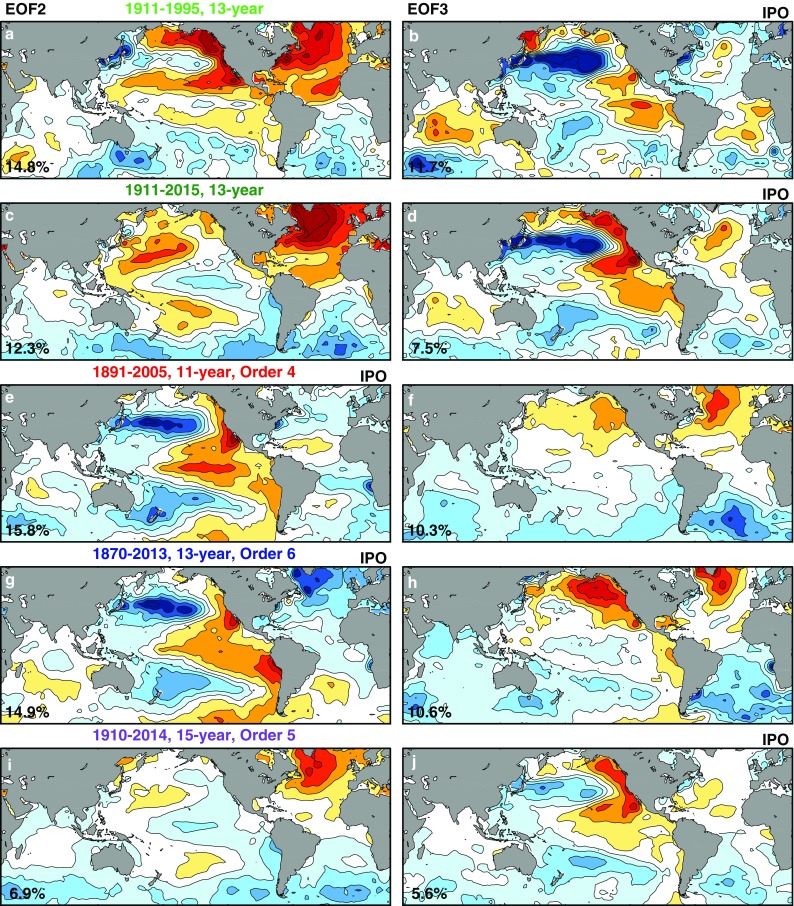
Reproducing IPOs. EOF decomposition of low-pass filtered SST. The left column of the spatial patterns shows the second EOFs and the right column the third EOFs. The first EOF, not shown, is the warming trend. The color code associates each EOF with its PC in Fig. 4. The percentage of variance explained by each mode is displayed in the lower-left corner of each EOF panel. From the top to bottom rows: (green): reproduction of Folland et al. (1999) for the period 1911–1995. (Darker green): extending Folland et al. (1999)’s time span to 1911–2015. (Red): reproduction of Parker et al. (2007). (Blue): reproduction of Henley et al. (2015). (Purple): for 15-year low pass filtered SST 1910–2014, to show the true interdecadal result. The choice of which of the EOF is IPO varies among authors, and is indicated in the top right of each panel

**Fig. 4:**
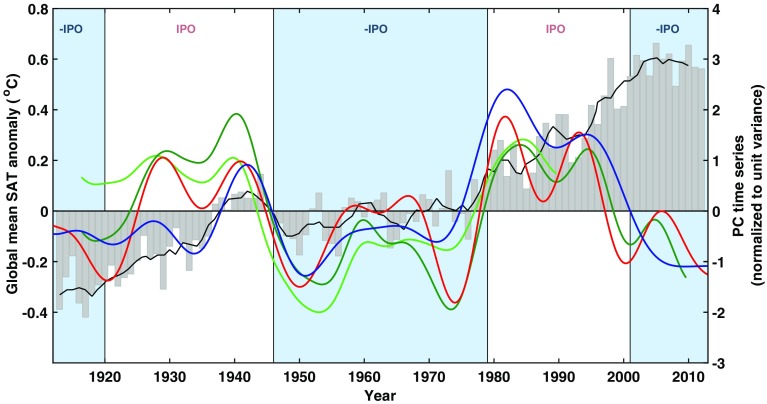
IPO time series. The time series, PCs, that correspond to IPO choice in Fig. 3 (either P_2_ or P_3_). The background in the two panels is from England et al. (2014), which shows the global-average surface air temperature anomalies for individual years as gray bars and a 5-year running mean as a black line. The positive and negative phases of low-pass filtered IPO index (Folland et al. 2002) are indicated by white and light blue shading, respectively. The PCs are normalized to have unit standard deviation with the scale indicated on the right-hand side. (Green): reproduction of Folland et al. (1999) for the period 1911–1995. (Darker green): extending Folland et al. (1999)’s time span to 1911–2015. (Red): reproduction of Parker et al. (2007). (Blue): reproduction of Henley et al. (2015)

In Figs. 3 and 4 we attempt to reproduce previous definitions of IPO using the same time spans and as close as possible their filter parameters. In the cases where such information is not available, we chose parameters to reproduce the spatial structure and time series as close as possible to the published ones. The details for each of the five cases are described in the Appendix in Table 1. Folland et al. (1999) used a 13.3-year low-pass filter in a SST record of 84 years. The Atlantic pattern in its EOF2 (Fig. 3a) resembles that for AMO in that there is an interhemispheric spatial pattern in the Atlantic with a stronger center of action in the North Atlantic, but there is another center of action in the northeastern Pacific so that there are two centers of action, as a result of what we called mode mixing. Its EOF3 (Fig. 3b) is PDO-like along with a weak asymmetrical pattern across the equator in eastern Pacific. Neither EOF is like ENSO in the tropical Pacific, possibly because the authors explicitly chose the parameters to remove tropical ENSO. There was concern that Folland et al. (1999)’s result may not be robust given its short time span. We therefore insert in the second row a case that is the same but with 20 more years of data: 1911–2015. The result is not significantly different. Additionally, the difference is almost unnoticeable if the filter is changed from 13.3- to 13-year low pass. Parker et al. (2007) used a 11-year low-pass filter; some decadal tail of ENSO variability remains in the tropical Pacific. However, if the tropical ENSO-related feature in Parker et al. (2007)’s case was due to the leakier filter (to allow in more higher frequencies), it is puzzling why a similar tropical pattern also exists in Henley et al. (2015)’s not so leaky 13-year low-pass filter used to obtain their EOF2. This will be reconciled later.

The positive and negative phase of IPO are shown in Fig. 4, as shadings upon the global-mean surface temperature, adapted from England et al. (2014). The PCs associated with the EOF that was chosen to be IPO for each of the four cases (using the same color code for the association) are superimposed. Although at first glance there is some similarity in the various time series shown, a closer examination reveals that the phase transitions often do not coincide with each other or with the “climate regime shifts” in the global-mean surface temperature. The reason that the time series are not more disparate than they are shown here is probably due to the fact that Henley et al. (2015) explicitly aimed to reproduce Parker et al. (2007)’s IPO index, who in turn followed Folland et al. (1999), that the shading in Fig. 4 was according to Folland et al. (2002), and that the two PCs (one light green and one dark green) are for the same case except one is for a longer period; these may give the appearance of similarity in the PCs. Furthermore, Chen and Tung (2017) showed that when globally averaged, IPO does not contribute to the global mean SST or land + ocean surface temperature. So this attribute of IPO with regards to its contribution to the variation of the global mean surface temperature is neither robust nor in fact effective.

It is known that IPO is sensitive to the details of processing because of the low degrees of freedom. We found that the degree of sensitivity can be ordered as: (i) sensitivity to *n*, the threshold of low-pass filter, (ii) sensitivity to data prior to 1910, (iii) sensitivity to differences in the Fourier filter used. For (iii), we mention that the choice of Fourier filter affects the choice of *n* if one wants to reproduce the historical spatial patterns. In the historical cases, most were done using Chebychev filter (adopted here also), with the exception of Folland et al. (1999)’s case, the details of which were not specified by the authors. We are able to reproduce the original EOF and PC using a Lanczos filter. Henley et al. (2015) mentioned that Folland et al.’s results can also be duplicated with a Chebychev filter.

**Fig. 5:**
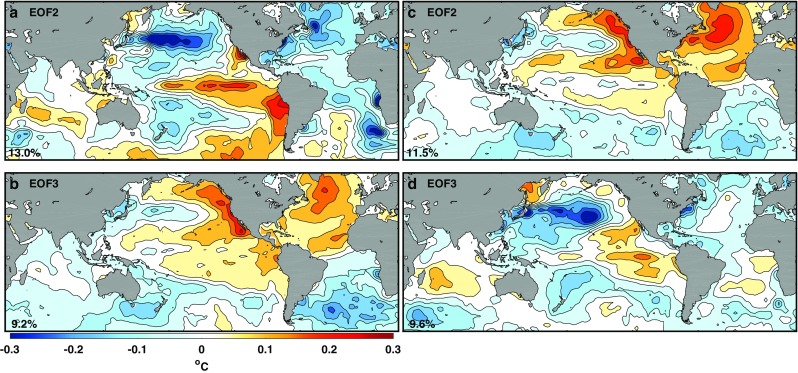
Sensitivity to nineteenth century data. Left panels: EOF2 and EOF3 of Parker et al. (2007)’s decomposition of 11-year low-pass filtered SST for the period 1891–2005. Right panels: similar to the left panels except using SST for the period 1910–2005

We now investigate (ii), sensitivity to time span. Changing time record length can affect the zero crossings of the PC, by 3–4 years in some cases and adding additional zero crossings in others. It however is understood that one should not expect to see a precise agreement in the zero crossings of decadally filtered data, since for such data the uncertainty is about a few years. The spatial structure can also be affected noticeably, but not qualitatively. For example, compare the first two rows in Fig. 3. They were obtained using the same filter and the same *n*, differing only in the time span by 20 years. On the other hand, a significant and qualitative change concerns whether SST data before 1910 are included in the time span. This is shown in Fig. 5. The puzzling tropical ENSO-related spatial pattern of Parker et al. (2007) shown in Fig. 3e was obtained for the period 1891–2005 using a 11-year low-pass filter. Using the same filter but applied to the period 1910–2005, any pattern that resembles ENSO largely disappears in the tropical Pacific (Fig. 5). Here we simply point out this sensitivity without attributing its cause, although the effect of data quality in the Pacific before the opening of the Panama Canal in 1914 needs to be further investigated.

Henley et al. (2015) used 13-year low-pass filter applied to the period 1870–2013. His EOF2 shown in our Fig. 3g also has a strong action center in the eastern tropical Pacific resembling ENSO. When the data before 1910 are removed, this feature in the eastern tropical Pacific also becomes very weak. This can be seen in Fig. 3c. For both cases, we see the dominance of AMO in the Atlantic, mixed with some PDO in the north Pacific.

Both the spatial pattern (in particular whether IPO has an ENSO-related but meridionally broader pattern in the eastern tropical Pacific) and the time series (whether its “regime shifts” coincide with that of the global-mean surface temperature) are sensitive to the type of filter used, the period analyzed and the preprocessing adopted (Henley et al. 2015). This is possibly due to the low degrees of freedom in the decadally low-pass filtered data. The second and third EOFs in this case form what North et al. (1982) called “effectively degenerate multiplets”, due to sampling errors. Note the small differences in the eigenvalues between the second and third EOFs in Fig. 3. With finite degrees of freedom this degeneracy occurs when the two eigenvalues overlap within the sampling error (Quadrelli et al. 2005; Wilks 2006). Those working in this area probably knew about the problem of sensitivity, which was often referred to as the “confounding” problem (Folland et al. 1999; Henley et al. 2015; Parker et al. 2007). However, the size of overlap of the eigenvalues has not previously been quantified. It turns out to be very large (Appendix Table 2), and so any amount of tuning or extending the period analyzed by a few decades will not solve the confounding problem.

It is apparent in reviewing the historical definition of IPO that there was a subjective choice involved: since the authors were looking for a “Pacific” pattern, if the second EOF (the leading dynamical mode) has a dominant center of action in the Atlantic, it is discarded, and the third EOF is chosen as IPO. Since the third mode is orthogonal to the second mode, this subjective choice eliminated the Atlantic center. However, given the effective degeneracy of these two modes, in reality these two modes are not distinct—since any linear combination of the two eigenvector is also an eigenvector—the Atlantic dominance cannot be removed or subjectively ignored.

Next, we systematically investigate (i), sensitivity to *n*. But first we need a mathematical framework for understanding the composition of the low-passed SST-based indices for any low-pass filter threshold *n*.

## EOF decomposition of unfiltered and low-pass filtered global SST

The dataset used in this study is NOAA’s ERSSTv3b SST (Smith et al. 2008), with a 3-month running mean done in the preprocessing. We shall refer to it as our “unfiltered” data in our presentation. The monthly mean data were used in CWT17, and very similar results were obtained, although the procedure involved one more rotation. By “filtering”, we specifically refer to multi-year low-pass filtering using a Fourier filter.

The unfiltered SST data is expressed in an orthogonal expansion of PCs in the form:

1 $$SST({\mathbf{x}},t)=\sum\limits_{{j=1}}^{\infty } {EO{F_j}({\mathbf{x}})P{C_j}(t)}$$

Both PCs and EOFs are orthogonal. The PCs are in addition normalized to have unit standard deviation.

### Rotated PC representation of unfiltered data

**Fig. 6:**
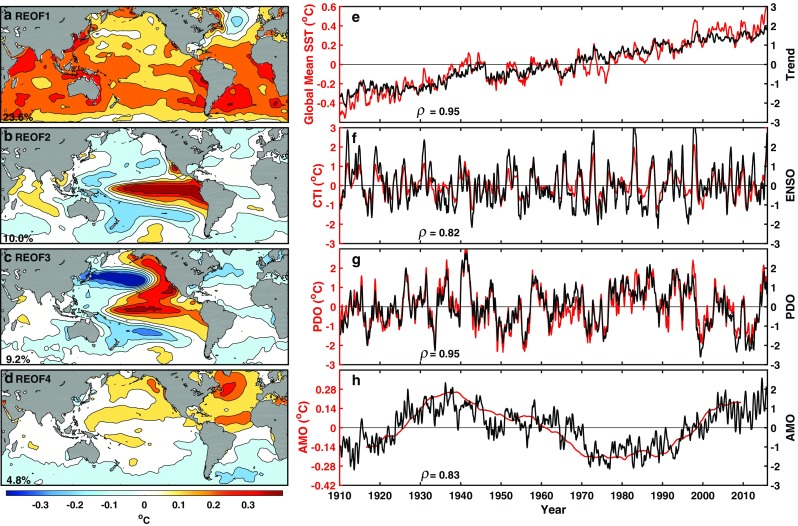
Rotated EOF of unfiltered SST. (From top to bottom) The first four EOF modes (the left column) and their corresponding PC time series (black curves on the right column with the scale indicated on the right) obtained from 3-month running mean SST of 1910–2015 with the seasonal cycle removed. No low-pass filter is applied before the EOF analysis. The percentage of variance explained by each mode is displayed in the lower-left corner of each EOF panel. Over a 1000 EOFs are used in calculating the percentage of variance. Superimposed on the PC time series from top to bottom are the global mean SST anomaly, the cold tongue index (Barnett 1984; Deser and Wallace 1987, 1990; Folland and Parker 1995; Zhang et al. 1997), the PDO index (Mantua et al. 1997) and AMO index (Enfield et al. 2001), respectively. The four indices are shown in red with the scale indicated on the left. The correlation coefficient between each PC and its corresponding index time series is indicated by $\rho$ inside each panel. All the four $\rho$’s are statistically significant at over 95% confidence level

A detailed justification for the choice of the general rotation angle between a pair of PCs can be found in CWT17. The pairwise rotated PCs are orthogonal (i.e. uncorrelated) and normalized, but the rotated EOFs are no longer orthogonal. PC1 now contains all the linear trends: the other PCs’ trends have been transferred to this rotated PC, following the convention of CWT and Huang et al. (1998) that the dynamical modes are oscillatory with zero trend. The result is shown in Fig. 6. A similar figure has been shown in CWT17.

**Fig. 7:**
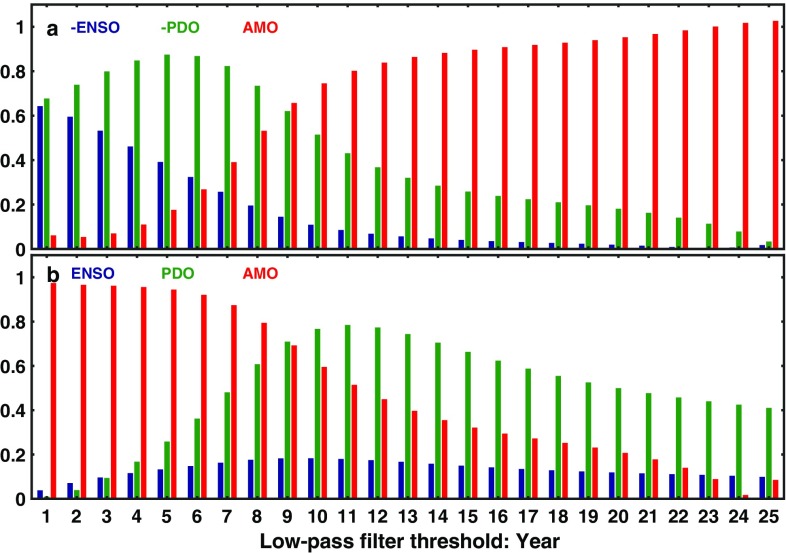
Composition of IPO in terms of the rotated PCs of the unfiltered SST. Top panel: *P*_2_, the leading PC after the trend in the EOF decomposition of the low-pass filtered data, as a function of *n* in years, the threshold of the low-pass filter. Blue bar indicates the proportion of the –PC2, the negative ENSO mode, as calculated using its coefficient ${\alpha _2}$ in Eq. (4). The green bar is ${\alpha _3}$ for –PC3, the negative PDO mode and the red bar is ${\alpha _4}$ for PC4, AMO mode. There is a small component due to PC1, the Trend mode, which is significant only at large *n*, and is not shown above. Higher PC’s (beyond 4) in the composition are not shown. They are generally negligible until *n* ~ 20. Bottom panel: same as Top panel but for *P*_3_ with positive ENSO and PDO modes

EOF2 is now the canonical ENSO, or variously called the Eastern Pacific ENSO, or ENSO-cycle mode. It has a large variance in the eastern Pacific and is more focused in the equatorial Pacific. More importantly there is very little variance in the extratropical Pacific. Its PC is dominated by the 2–7 years interannual frequencies (as can be seen by how this mode behaves under filtering, in Fig. 7 below), and highly correlated with the Cold-Tongue Index (r = 0.82). In the conventional EOF decomposition, this almost monopole spatial mode is not mathematically permitted because it cannot be made orthogonal to the global warming mode (EOF1). It now exists in the rotated EOF as a mode because we no longer require spatial orthogonality.

Our global EOF3 is very close to the regionally defined PDO; its PC has a broad spectrum and appears to be red noise below 10 years and white noise above 10 years (CW16; Newman et al. 2016). Its correlation with the PDO index of Mantua et al. (1997) is very high (r = 0.95). Its spatial structure north of 20°N is close to the regionally defined PDO, but this global version has an extension of the opposite sign into the central equatorial Pacific of weaker amplitude. It is much weaker in the eastern Pacific, near the coast, reminiscent of Folland et al. (1999)’s description of IPO in the pan-Pacific region. It is also similar to the leading EOF of 6-year low-passed filtered pan-Pacific SST* shown in Fig. 1. EOF4 is AMO, with a multidecadal frequency range (r = 0.83).

The rotation of the PCs does not change the representation of the SST field expressed in Eq. (1), but gives it a better physical interpretation as the rotated PCs have largely distinct frequency ranges, and the EOFs, though global, have the familiar spatial patterns from previous regional definitions. We shall call the first EOF the trend mode, the second ENSO mode, the third PDO and the fourth AMO.

### Effect of low-pass filtering

Another way to isolate the low-pass part of the variability is to perform a low-pass filtering. We now apply an *n*-year low-pass filter, denoted by [ ], to both sides of Eq. (1). The derivation below is the same whether the PCs are rotated or not:

2 $$[SST({\mathbf{x}},t)]=\sum\limits_{{j=1}}^{\infty } {EO{F_j}({\mathbf{x}})[P{C_j}(t)]}$$

The filtered PCs are no longer orthogonal. They are also no longer normalized as filtering reduces their variance. For presentation purpose, we renormalize the filtered PCs by their respective standard deviation, and the normalization constant is absorbed into the EOFs.

The low-pass filtered data on the left-hand side is expanded in a different, but conventional, orthogonal EOF expansion, following tradition. We denote its EOF by *E* and its PC by *P*. Both *E* and *P* are orthogonal. *P* is in addition normalized to unit standard deviation

3 $$[SST({\mathbf{x}},t)]=\sum\limits_{{i=1}}^{\infty } {{E_i}({\mathbf{x}}){P_i}(t)}$$

IPO may either appear as *P*_2_, or *P*_3_. The choice is not clear-cut. In the presence of closely spaced eigenvalues, sometimes the choice is subjective, often being dependent on the authors’ preference. The current convention is to define *P*_2_ as the IPO.

The PCs of the filtered SST can be obtained by taking the spatial inner product 〈.〉 of ${E_m}$ on both sides of Eqs. (3) and (2), recalling that the $E$’s are orthogonal, as:

4 $${P_m}(t)=\sum\limits_{{j=1}}^{\infty } {{\alpha _j}[P{C_j}(t)]}$$

$${\alpha _j}=\frac{{\left\langle {EO{F_j}({\mathbf{x}}) \cdot {E_m}({\mathbf{x}})} \right\rangle }}{{\left\langle {{E_m}({\mathbf{x}}) \cdot {E_m}({\mathbf{x}})} \right\rangle }}$$

where ${\alpha _j}$ can be interpreted as the spatial projection of the unfiltered EOF onto the filtered EOF, i.e. $EO{F_j}$ onto ${E_m}$, normalized by the variance of the latter. On the other hand, ${\alpha _j}$ cannot be obtained by projecting the filtered PC (${P_m}$) onto the unfiltered PC after the latter have been filtered, due to the fact that the terms in the sum on the right side of Eq. (4) are not orthogonal. Nevertheless, Eq. (4) is in a form similar to multiple linear regression of the filtered PC using the set of “predictors” on the right hand side. The purpose of going through the derivation above is to systematically derive these predictors and show that the set of the predictors is complete, despite them being non-orthogonal. Since Eq. (4) is exact, the complete composition of the filtered PC can be calculated. This is theoretically different than “regression” or “projection”. In the latter procedures one does not know if a component is missing. Nevertheless, it turns out that only the first four terms are significant for decadally filtered SST; so only these are shown.

We can also obtain the component of the filtered EOF. Taking the inner product of ${P_m}$ on both sides of Eqs. (3) and (2), and recalling that the P’s are orthogonal and has unit variance, we find:

5 $${E_m}=\sum\limits_{{j=1}}^{\infty } {EO{F_j} \cdot \left\langle {[P{C_j}] \cdot {P_m}} \right\rangle }$$

Globally averaging both sides of Eq. (5) then yields the global-mean components of IPO for either m = 2 or 3.

Figure 7a shows how the components of *P*_2_ change as a function of *n* (in years), the low-pass filter threshold, according to the right-hand side of Eq. (4). Figure 7b shows *P*_3_. For small *n*, the mixed ENSO and PDO modes dominate *P*_2_, while AMO mode dominates *P*_3_. Since AMO’s frequency is multidecadal, it is little affected by the low-pass filtering, but ENSO mode is greatly affected and decreases to less than half that of the PDO for n > 5. At or above *n* = 10, the composition of *P*_2_ is given mostly by AMO. That is, after low-pass filtering the SST, the leading EOF (after the warming trend) is dominated by an Atlantic pattern. The tropical ENSO-related spatial pattern commonly associated with IPO largely disappears for decadally filtered SST. P_3_ is a mixture of AMO and PDO. AMO dominates below n = 9 while PDO contributes more than 50% as much as AMO for n > 11 years.

**Fig. 8:**
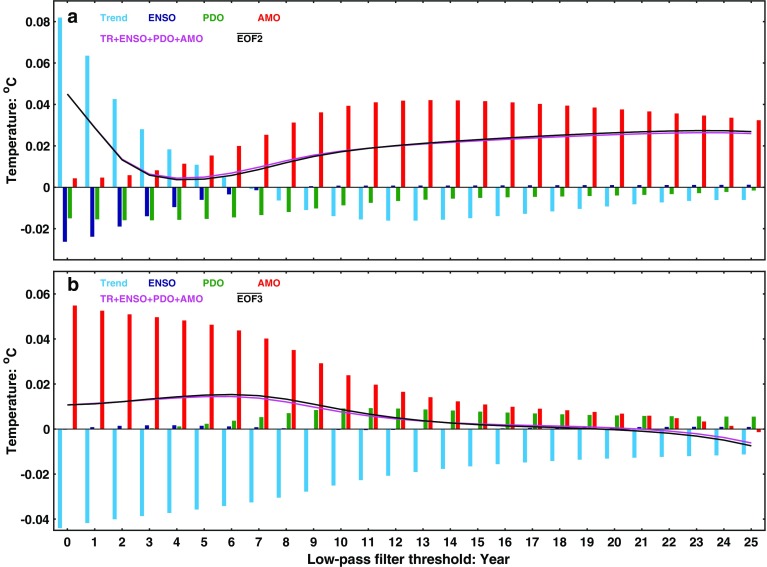
Composition of the global mean of IPO in terms of the rotated EOFs of the unfiltered SST. The global mean of IPO’s EOF and its components. The black curve is the global mean of IPO, as either the second (top panel) or third (lower panel) EOF of the low-pass filtered SST. The magenta curve is the sum of the first four modes, showing that the global mean of IPO is almost entirely given by this partial sum. The red bar is the component contributed by AMO, the green by PDO, the blue by ENSO and the cyan the trend mode

As a measure of the composition of IPO’s EOF, Fig. 8 shows the global mean of IPO in terms of the global means of the rotated EOFs according to Eq. (5). The top panel shows the global average of *E*_2_. It is seen to be dominated by AMO. The bottom panel shows the global average of *E*_3,_ which is practically zero for decadally filtered SST. When the third EOF is chosen as IPO to emphasize its Pacific variability, as in Folland et al. (1999), IPO has an almost zero global-mean SST because of the dominance of PDO in its composition. The contribution of PDO to the global mean SST and the global mean surface temperature (including the effect of teleconnection to the continents) is studied in Chen and Tung (2017). It was found that the PDO’s contribution to the global mean surface temperature is one order of magnitude less than that by AMO.

## Alternative definition of IPO

As an alternative to using EOF analysis of the low-passed SST to define IPO, Henley et al. (2015) proposed using the difference of SSTs in three regions in the Pacific to define an IPO Tripole Index (TPI): $TPI={T_2} - 0.5({T_1}+{T_3})$.

The three regions are: *T*_2_ the central and eastern equatorial Pacific (10°S–10°N, 170°E–90°W), *T*_1_ the Northwest Pacific (25°N–45°N, 140°E–145°W), and *T*_3_ the Southwest Pacific (50°S–15°S, 150°E–160°W). The index is filtered using a 13-year low-pass filter to mimic IPO index. The unfiltered TPI index (Henley et al. 2015) consists mainly of ENSO and the PDO (not shown). The low-pass filtering greatly reduces ENSO component, leaving mostly the PDO, which has an almost zero global mean. This has been discussed in Chen and Tung (2017).

## Conclusion

IPO is often referred to as an interdecadal variability originating in the tropical Pacific that has important global influence. It is commonly thought that its spatial pattern is like ENSO but broader meridionally, and it modulates the interannual ENSO over interdecadal time scales. It is defined by an EOF analysis of low-pass filtered SST data. Without the filtering, it is shown here that the leading variability in the Pacific is the interannual ENSO cycle, and PDO in the North Pacific, which is decadal but its spectrum has broad tails. In the Atlantic, there is a clear AMO, with a multidecadal time scale. IPO is not a distinct climate variability, but is composed of these three forms of variability in various proportions depending on the degree of filtering. Such a combination is in the form of linear superposition.

As the second EOF (the leading dynamical mode) of the decadally filtered SST, IPO is mostly AMO. So the contribution to the global mean SST variability in the multidecadal scale often attributed to IPO in the Pacific is actually by AMO in a different ocean basin, the Atlantic. If the third EOF of decadally low-pass filtered SST is defined as IPO, as originally defined by Folland et al. (1999) and Power et al. (1999) IPO contributes almost zero to the global mean SST. The Pacific contributes to the global-mean SST strongly in the 2–7 years timescale, which are however not present in low-pass filtered data.

There is a well-defined decadal variability in the Pacific in the form of PDO. The pan-Pacific version was shown in CW16. It can be obtained through rotated PCs without low-pass filtering and so does not have the problem with loss of degrees of freedom. The description of IPO by the original authors (Folland et al. 1999; Power et al. 1999) probably was referring to the pan-Pacific PDO. Its impact on the global-mean SST, however, is substantially weaker than AMO, by one order of magnitude.

## Appendix: Method

### The principal component analysis (PCA)

Monthly ERSST.v3b 2° × 2° global sea surface temperature (SST) data since 1854 have been used to derive the principal components (PCs) and the empirical orthogonal functions (EOFs). This dataset was previously used by other authors in deriving IPO. The purpose of this section is to repeat as closely as the previous work and show the sensitivity of the results. At each grid point, we take the 3-month running mean with seasonal cycle removed. Certain low-pass filter is applied to the resultant time series at each grid point (see Table 1 and text below).

**Table 1:** The data span and the filtering parameters of the low-pass filter used to generate Figs. 3 and 4

	Data span	*n*	Γ_*c*_ (years)	Filter
Case 1 (green)	1911–1995		13.3	Lanczos
Case 2 (dark green)	1911–2015		13	Lanczos
Case 3 (red)	1891–2005	4	11	Chebyshev
Case 4 (blue)	1870–2013	6	13	Chebyshev
Case 5 (purple)	1910 --2014		15	Lanczos

The corresponding color codes are in the parentheses

### Spectral filtering

Folland et al. (1999)’s filter was not specified, while Parker et al. (2007) and Henley et al. (2015) used the Chebyshev filter. We therefore used Chebyshev type I low-pass filter in all calculations except that we chose Lanszos filter for Folland et al. (1999)’s case. We used the Matlab function cheby1, which requires four input parameters: the order of the Chebyshev filter *n*, the decibels of peak-to-peak passband ripple *R*_*p*_, the normalized passband edge frequency *W*_*p*_, and the type of the filter. We have specified the low-pass filter (i.e. input as **‘**low’) as the type of the filter. We set ${R_p}= - 20{\log _{10}}\frac{1}{{\sqrt 2 }}$ as the half power and ${W_p}=\frac{1}{{{f_N}{\Gamma _c}}}$, where ${\Gamma _c}$ is the cut-off period (e.g., 11 years) and ${f_N}$ is the Nyquist frequency (0.5 cycle per year). The details are shown in Table 1.

### The uncertainty of the EOFs

The principal component analysis is based on a sample covariance matrix of a finite dataset. Thus the resultant eigenvalues (i.e. variance explained, λ) and eigenvectors (i.e. EOFs) are subject to sampling uncertainties, which can be obtained by the perturbation theory (North et al. 1982; Quadrelli et al. 2005). Given multiple observations of ${N_{eff}}$ “effective” temporal points and *M* estimated EOFs, the uncertainties to first order in $N_{{eff}}^{{ - 1/2}}$, associated the *i*-th EOF ${\Phi _i}$ that explains a fraction ${\lambda _i}$ of the total variance are, respectively,

6 $$\delta {\Phi _i}=\frac{1}{{\sqrt {{N_{eff}}} }}\sum\limits_{\begin{subarray}{l} j=1 \\ j \ne i \end{subarray} }^{M} {\frac{{\sqrt {{\lambda _i}{\lambda _j}} }}{{{\lambda _i} - {\lambda _j}}}{\Phi _j} \cdot {w_{ij}}}$$

7 $$\delta {\lambda _i}={\lambda _i}\sqrt {\frac{1}{{{N_{eff}}}}} \cdot {w_{ii}}$$

where the ${w_{ij}}$ are uncorrelated unit normal random perturbations. In this work, the upper and lower bounds of the $(1 - \alpha ) \times 100\%$ confidence interval of ${\lambda _i}$ are given by$\frac{{\hat {\lambda }}}{{1 \pm {z_{1 - \alpha /2}}\sqrt {\frac{2}{{{N_{eff}}}}} }}$, where $\hat {\lambda }$ is the *i*-th estimated eigenvalue and ${z_{1 - \alpha /2}}$ is the value of the standard score at the $\left( {1 - \frac{\alpha }{2}} \right) \times 100\%$ percentile of the two-tailed standardized normal distribution (Wilks 2006). We assume ${N_{eff}}$ to be the total number of years in our calculation. Results are shown in Table 2.

**Table 2:** The estimated percentage of variance explained by each of the first three EOF modes and its 95% confidence interval (CI) for the five cases shown in Fig. 4

	${\hat {\lambda }_1}$ (%)	95% CI	${\hat {\lambda }_2}$ (%)	95% CI	${\hat {\lambda }_3}$ (%)	95% CI
Case 1	49.6	[38.1%, 70.9%]	14.8	[11.4%, 21.1%]	11.7	[9.0%, 16.7%]
Case 2	58.4	[46.0%, 80.6%]	12.3	[9.7%, 16.9%]	7.5	[5.9%, 10.3%]
Case 3	35.0	[27.8%, 47.2%]	15.8	[12.5%, 21.3%]	10.3	[8.2%, 14.0%]
Case 4	39.0	[31.7%, 50.7%]	14.9	[12.1%, 19.4%]	10.6	[8.6%, 13.7%]

There is always an overlap between the 95% confidence intervals of the second and the third modes for all four cases, which implies that these two modes are statistically indistinguishable. The number of the years N is taken as the sample size in calculating the uncertainties, a gross overestimate. The effective sample size should be an order of magnitude less than the number of years for the low-pass filtered SST data used in the EOF analysis. So the true 95% CIs must be wider, although it is difficult to estimate them accurately

### Effectively degenerate multiplets of EOFs

Equation (6) reveals that the uncertainty of the spatial EOF critically depends on the mutual differences of ${\lambda _i}$ from all other eigenvalues: $\delta {\Phi _i}$ will be large if there exists one eigenvalue ${\lambda _j}$ that is very close to ${\lambda _i}$. Mathematically, this means that if ${\lambda _i}={\lambda _j}$, any linear combination of Φ_*i*_ and Φ_*j*_ is also a spatial EOF corresponding to ${\lambda _i}={\lambda _j}$ and thus the spatial EOF is physically indeterminate. Statistically ${\lambda _i}$ and ${\lambda _j}$ may be indistinguishable within the error bounds $\delta {\lambda _i}$ and $\delta {\lambda _j}$, even if they are not the same. In these cases, ${\Phi _i}$ and ${\Phi _j}$ might be mixed (North et al. 1982; Wilks 2006), making the physical interpretation difficult. These statistically indistinguishable modes have been known as “effectively degenerate multiplets” (North et al. 1982).

The width of the confidence interval of ${\lambda _i}$, which depends on ${N_{eff}}$, is critical to determining which pairs of the EOFs may be degenerate. If a 10-year low-pass filter is applied, one may expect that ${N_{eff}}$ is of order *N*/10 but some rigorous calculation taking into account autocorrelation of the low-pass filtered data suggests an even smaller value of ${N_{eff}}$ (Bretherton et al. 1990). For our purpose it suffice to show that the overlap exists for all the cases shown in Fig. 3 even if we use a gross overestimate of ${N_{eff}}$ as the total number of years, *N* ~ 100. This results in the most conservative estimates of the confidence intervals of ${\lambda _i}$, since using a smaller value of ${N_{eff}}$ would result in an even wider confidence interval.
